# Oscillatory Underpinnings of Mismatch Negativity and Their Relationship with Cognitive Function in Patients with Schizophrenia

**DOI:** 10.1371/journal.pone.0083255

**Published:** 2013-12-17

**Authors:** Muzaffer Kaser, Fruzsina Soltesz, Phil Lawrence, Sam Miller, Chris Dodds, Rodney Croft, Robert B. Dudas, Rashid Zaman, Emilio Fernandez-Egea, Ulrich Müller, Anna Dean, Edward T. Bullmore, Pradeep J. Nathan

**Affiliations:** 1 Department of Psychiatry, University of Cambridge, Cambridge, United Kingdom; 2 Bahcesehir University, Istanbul, Turkey; 3 Behavioural and Clinical Neuroscience Institute, University of Cambridge, Cambridge, United Kingdom; 4 GlaxoSmithKline, Clinical Unit Cambridge, Medicines Discovery and Development, Cambridge, United Kingdom; 5 Department of Psychology, University of Wollongong, Wollongong, Australia; 6 Cambridge and Peterborough NHS Foundation Trust, United Kingdom; 7 South Essex Partnership NHS Foundation Trust, United Kingdom; 8 Brain Mapping Unit, Department of Psychiatry, University of Cambridge, Cambridge, United Kingdom; 9 School of Psychology and Psychiatry, Monash University, Melbourne, Australia; 10 Neuroscience Discovery Medicine, UCB Pharma, Brussels, Belgium; University of Queensland, Australia

## Abstract

**Background:**

Impairments in mismatch negativity (MMN) generation have been consistently reported in patients with schizophrenia. However, underlying oscillatory activity of MMN deficits in schizophrenia and the relationship with cognitive impairments have not been investigated in detail. Time-frequency power and phase analyses can provide more detailed measures of brain dynamics of MMN deficits in schizophrenia.

**Method:**

21 patients with schizophrenia and 21 healthy controls were tested with a roving frequency paradigm to generate MMN. Time-frequency domain power and phase-locking (PL) analysis was performed on all trials using short-time Fourier transforms with Hanning window tapering. A comprehensive battery (CANTAB) was used to assess neurocognitive functioning.

**Results:**

Mean MMN amplitude was significantly lower in patients with schizophrenia (95% CI 0.18 - 0.77). Patients showed significantly lower EEG power (95% CI -1.02 - -0.014) in the ~4-7 Hz frequency range (theta band) between 170 and 210 ms. Patients with schizophrenia showed cognitive impairment in multiple domains of CANTAB. However, MMN impairments in amplitude and power were not correlated with clinical measures, medication dose, social functioning or neurocognitive performance.

**Conclusion:**

The findings from this study suggested that while MMN may be a useful marker to probe NMDA receptor mediated mechanisms and associated impairments in gain control and perceptual changes, it may not be a useful marker in association with clinical or cognitive changes. Trial-by-trial EEG power analysis can be used as a measure of brain dynamics underlying MMN deficits which also can have implications for the use of MMN as a biomarker for drug discovery.

## Introduction

Schizophrenia has conventionally been defined by positive symptoms (i.e. hallucinations or delusions) and negative symptoms (i.e. avolition, social withdrawal). However, it has increasingly been recognised that cognitive impairments in schizophrenia represent a third dimension which has deleterious effects in the majority of patients [1]. Cognitive impairments in various domains have been well documented in patients with schizophrenia [2] and are associated with functional outcome [3]. Improving cognition in schizophrenia is of paramount importance to help improve the patients’ quality of life. Although currently available antipsychotic medication can help to alleviate positive and negative symptoms, they do not address cognitive symptoms sufficiently. Therefore, there has been a shift in drug development research for schizophrenia towards new strategies to tap into cognitive impairment. CNTRICS (Cognitive Neuroscience Treatment Research to Improve Cognition in Schizophrenia) initiative, an international network of researchers reported that evaluation tools to monitor cognitive impairment in schizophrenia were crucial. According to a consensus report by CNTRICS, mismatch negativity (MMN) was classified as one of the measures of gain control and it was regarded as a suitable marker to detect perceptual impairments in schizophrenia [4] which renders it suitable for testing pro-cognitive effects of drugs in development for cognitive impairment in schizophrenia 

MMN is an event related potential (ERP) reflecting pre-attentive detection of auditory changes in response to deviant or novel stimuli [5]. Neural processes generating MMN are suggested to reflect brain responsiveness to salience induced by sensorial change relative to memory expectations [6]. After the first report on deficits in MMN generation in schizophrenia [7], numerous studies have consistently showed smaller MMN amplitudes in patients with schizophrenia [8,9]. The majority of studies have found no association between MMN amplitude and clinical symptoms [10]. MMN deficits are not affected by antipsychotic medication [11,12]. On the other hand, NMDA antagonists have been shown to reduce MMN amplitude in humans [13] and in rats [14]. There is a range of paradigms (using frequency, duration or phoneme deviants) used to generate MMN. A roving paradigm was used by studies in healthy volunteers [15] and relatively fewer studies in schizophrenia [16]. Data have shown that MMN generation with the roving paradigm is consistent to that of the oddball paradigm. Clinically, duration MMN has been related more with the disease and prominent from early stages of schizophrenia [8,17]. Duration MMN reductions are relatively stable through disease course and are associated with poorer social functioning over time [18,19]. MMN has high test re-test reliability [20] and it does not require voluntary attention, thus the confounding effects of motivational factors are minimized. A few studies reported reduced MMN amplitudes in first degree relatives of patients [21,22], suggesting its potential as a neurophysiological endophenotype. But also some studies reported no reduction in relatives [23]. 

Previous studies investigating the relationship between MMN deficits and neuropsychological performance led to contradictory results as some studies indicated correlations between MMN deficits and particular cognitive domains (i.e. executive function [24] or memory [16]), while others did not [25]. CANTAB (Cambridge Automated Neuropsychological Test Assessment Battery) has good test-retest reliability and it has been widely used in treatment studies of schizophrenia [26]. Neural bases of the cognitive domains identified in CANTAB are well established [27] and the CANTAB schizophrenia battery was developed based on translational utility of each test and their known sensitivities to pharmacological manipulation [28].

Despite the substantial body of evidence suggesting that neural oscillations underlie cognitive processes [29] and that ERPs are composed of neural oscillations [30], there has been only a couple of studies investigating the oscillatory activity underlying the MMN deficits in schizophrenia [31,32]. The former indicated that patients lacked theta power in MMN paradigm [32], and the latter reported that alterations in delta and theta power in patients with schizophrenia were correlated with MMN amplitude [31]. However their analyses eliminate some trial-by-trial fluctuations in power and don't allow the investigation of phase-locking [31]. Previous research with healthy volunteers indicated the role of oscillatory power modulation and phase coherence at theta frequency band as reflecting the underlying MMN processes at the spectral level [33,34]. Specifically, event-related spectral perturbations [35] (ERSPs; power) can provide a more sensitive measure of underlying cognitive processes as they can detect alterations in the power of neural oscillations across several frequency bands and on a trial by trial basis, which otherwise would not be captured by the averaged time domain waveforms [36]. On the other hand, the inter-trial phase coherence [37] (ITC; coherence or phase locking), is a sensitive index for the phase coherence of neural oscillations across individual trials; information which also vanishes from the averaged time domain waveforms [38]. For example, an increase in oscillatory power results in an increase in the amplitude of time domain average waveform, but so too does the increase in phase alignment, with the power of the neural oscillation itself being unchanged [36]. Therefore, time-frequency power and phase analyses provide a more sensitive and detailed picture of brain dynamics during MMN, capable of separating independent neural processes and might provide more sensitive markers of the altered neural circuits involved in MMN processing in schizophrenia. 

The objective of the current study was to 1) explore the underlying oscillatory activity of MMN in patients with schizophrenia compared to healthy controls by time-frequency analyses and 2) investigate the relationship between MMN (i.e. MMN amplitude, power (ERSP) and phase-locking) and neurocognitive functioning. Investigating the spectral components of MMN might help to develop refined and more sensitive biomarkers for the effects of novel treatments in schizophrenia. This study provides the first data on the exploration of power and coherence components of MMN in patients with schizophrenia. Secondly, the relationship between MMN parameters and neurocognitive functioning (as well as clinical measures) and functional outcome was investigated. 

## Methods

The study sample included 21 right handed patients (17 male, 4 female) aged between 18-55 and diagnosed with schizophrenia or schizoaffective disorder (only one patient) according to DSM-IV using Mini International Neuropsychiatric Inventory (MINI) assessment [39]. Patients had no other axis I disorders, and had been clinically stable for 3 months. Antipsychotic medications were not altered for at least the last 2 months and doses were not altered for at least 1 month prior to enrolment. Exclusion criteria included alcohol/substance abuse (except nicotine for patients), head trauma, mental retardation and failure to comply with study procedures. All participants had urine drug screening prior to testing. 

Control subjects (14 male, 7 female) were age (years) and education matched, healthy, non-smoker volunteers with no history of mental disorder. Healthy subjects were screened for axis I psychiatric disorders using the MINI. None of the control participants had first or second degree relative with schizophrenia. The groups were comparable with regards to age, gender and premorbid IQ, whilst control subjects had higher average current IQ scores than patients (Table 1). For schizophrenia patients, parental education years were taken as an index of educational level, which was comparable between groups (Table 1). Clinical characteristics of patients are presented in Table 2. 

**Table 1:** Demographic characteristics of patients and controls.

	Patients (n=21)	Controls (n=21)	Statistics (t and df values)	95% Confidence Intervals	p value
Age	34.19 (10.91)	31.52 (9.13)	t = - 0.859 df = 40	- 8.943 - 3.610	0.39
Education (years)*	12.96 (3.26)	14.95 (2.45)	t = 1.995 df =31	-0.044 - 4.021	0.055
Premorbid IQ	110 (6.96)	112.19 (5.02)	t = 1.159 df = 39	-1.633 - 6.014	0.25
Current IQ	96.21 (14.85)	109 (10.25)	t = 3.195 df = 38	4.685 - 20.894	0.01

^*^ Education (years) for patients stands for the parental education years.

**Table 2:** Clinical characteristics of patients.

	Minimum	Maximum	Mean	SD
Medication (mg)* (Chlorpromazine equivalent dose)	75	400	247.19	108.18
Medication duration (years)	0.3	10	3.81	3.11
BPRS	2	29	12.90	8.38
PANSS-Positive	7	22	12.43	4.54
PANSS-Negative	8	30	14.81	6.57
PANSS-General Psychopathology	16	35	23.19	5.75
PANSS-Total	31	79	50.80	14.08
BDI	0	63	13.57	15.12
BAI	0	28	8.52	8.30
WSAS	1	36	16	11.17

^*^ All patients were on monotherapy of second generation antipsychotics; risperidone long-acting (n=5), risperidone oral (n=1), clozapine (n=8), olanzapine (n=5), quetiapine (n=1), amisulpiride (n=1).

This study was a part of a larger study (United Kingdom Clinical Research Network Portfolio ID: 7470) sponsored by GlaxoSmithKline Pharmaceuticals. The procedure was conducted in accordance with "good clinical practice" (GCP), and the guiding principles of the Declaration of Helsinki. The study was subject to Independent Ethical Committee review and was approved by Cambridgeshire 3 Research Ethics Committee on 24^th^ July 2008 (REC reference 08/H0306/52). Written informed consents were obtained for each subject before participation in the study in line with GCP. 

### Clinical Measures

1 Positive and Negative Syndromes Scale [40]: A semi-structured interview scale addressing symptom severity in three main clusters: positive symptoms, negative symptoms, and a general psychopathology subscale.
2 Brief Psychiatric Rating Scale [41]: BPRS is used to evaluate the severity of psychotic symptoms. It is composed of eighteen items scored on a 7 point range. The total BPRS score was used.
3 Beck Depression Inventory-II [42]: BDI-II is a self-report scale composed of 21 items each covering a 4-point range for the severity of depressive symptoms.
4 Beck Anxiety Inventory [43]: BAI is a 21-item self-report questionnaire used to evaluate anxiety symptoms over the last week. Each item is scored on a range between 0-3 to assess level of discomfort.
5 Wechsler Abbreviated Scale of Intelligence [44]: The WASI uses the vocabulary, similarities, block design and matrix reasoning subtests of the WAIS-III to provide an estimate of full scale IQ. WASI has been shown to be a reliable reflection of WAIS-III scores and has the advantage of shorter testing time.
6 National Adult Reading Test [45]: The NART is used to assess reading ability of words with irregular spelling. Ability to pronounce such words is preserved across a range of neurocognitive disabilities and is thus indicative of premorbid intelligence.
7 Work and Social Adjustment Scale [46]: The WSAS is a validated measure of self-reported functional impairment. Used to assess impairment in various areas of daily living such as the ability to work, home management, social and private leisure activities, and ability to form and maintain close relationships. Each item is scored between 0-8 and higher points denote more disability. In this study total WSAS score was used.
8 Neuropsychological Measures: A selection of six tests from the CANTAB battery addressing five key cognitive domains in schizophrenia was used. These included Spatial Working Memory (SWM); Intra/Extra Dimensional Set Shift (IED); One Touch Stockings of Cambridge (OTS); Paired Associates Learning (PAL); Rapid Visual Processing (RVP) and Emotional Faces Recognition (ERT) tests. The details of the CANTAB tests can be found at http://www.cantab.com/cantab-tests.asp [47].

### MMN Recording and Analysis

Subjects were seated comfortably in front of a CRT monitor and asked to watch scenes from a wildlife documentary without sound. Tones were played binaurally through ear inserts at 80 dB. Duration deviants with roving frequency design were used to generate MMN. Standard tones were 50 ms and deviant tones were 100 ms, with the latter presented at the end of each train of standard tones of 3-15 in length. Between each train of standard/deviant stimuli, one (50%) or two (50%) new standard tones were presented. These latter tones served as masks, and were standard tones that differed in frequency to those in both the preceding and subsequent train. Each train was presented at a different frequency, separated from the previous train by at least 500 Hz (tone range 100 - 5000 Hz). The task was divided into 6 blocks, such that each block contained 1 of each of the 11 trains with 1 intervening mask, plus 1 each of the 11 trains with 2 intervening masks, all randomly presented, and stimulus onset asynchronies randomly assigned (from 0.35 to 0.45 seconds). The overall possibility of deviant tones was approximately 15%. Although the paradigm used in this study is relatively novel, for this particular paper the memory trace effects of the standards were not examined. Thus, the paradigm used is basically a normal duration deviant except that the standard memory trace is restarted for each train [15,16].

Data were collected using a Neuroscan SymAmps2 acquisition system at 24bit resolution, 1000 samples per second from 64 channels, with additional electrodes placed around the eyes to capture activity from corneal-retinal potentials in the eyes (electro-occulogram) and on the nose. Electrode impedances were below 10 kOhms at the start of the recording. Data were recorded online in AC mode with a 0.5 Hz to 100 Hz bandpass filter. Offline data were re-referenced to the nose electrode and a second-order lowpass (80 Hz) Butterworth filter (corresponding to 12 dB/octave rolloff) has been applied. (For the time-domain ERP analysis the data was further filtered with a 30 Hz lowpass zero phase shift filter in order to remove high-frequency noise from the MMN wave. This filtering does not affect the time-frequency analyses). Data were then epoched into 600ms segments (-100 ms to +500 ms around event markers), baseline corrected (relative to the pre-stimulus interval) and epochs containing activity of more than +/- 100µV at any scalp site were discarded [48]. Further data processing was done using Matlab (MathWorks).

### Time-domain ERP analysis

Responses to standard stimuli at all train positions (with the exception of position 1) were then averaged together to create the standard average wave. Responses to deviant stimuli were averaged to create the average deviant wave. The standard was then subtracted from the deviant response to create the difference waveform. The MMN waveforms were checked for appropriate scalp topography (fronto-central negativity) and the presence of the polarity reversal at the mastoid electrodes (positive) relative to Fz (negative). Independent sample point-by-point t-tests within the 150-250 ms poststimulus window [34] across all electrodes were also run in order to confirm the temporal location of the MMN group difference. The p-values of multiple point-by-point t-tests were corrected for false discovery rate (FDR) utilizing the method described by Benjamini and Yekutieli [48]. The alpha was set to 5%, and results were deemed significant if the probability of type I error (false positives) was also lower than 5%. After confirming (with the above described point-by-point statistics) that the MMN group difference emerged between 170-210 ms (adjusted p<0.036 for all points) over the fronto-central electrodes, the mean amplitude of this significant interval and the MMN peak latency at the Fz electrode site were extracted and subjected to a two-sample t-test comparing the two groups. The same procedures were applied on the MMN waveforms over the two mastoid electrodes. Furthermore, besides the difference wave, ERPs in response to the standard and deviant stimuli from the Fz electrode were also examined. Mean amplitudes from the time window of the significant MMN peak (170-210 ms) have been submitted to a Group × Condition mixed-design ANOVA in order to reveal whether there were significant group differences separately in the standard and/ or deviant waveforms.

### Time-frequency EEG analysis

Following up the time-domain MMN analysis, time-frequency power and phase-locking (PL) analyses were performed on all trials using short-time Fourier transforms with Hanning window tapering, resulting in a time-frequency landscape with a resolution of 8 ms in time and 0.49Hz (from 0.5 to 30Hz) in frequency. For the time-frequency analysis scripts from the EEGLAB toolbox [37] were used. Three thousand milliseconds epochs were used for the decomposition. “Regions of interest” of the whole time-frequency landscape in the time interval of the MMN ERP component for the group comparisons were then selected based upon exploratory 2-tailed point-by-point t-tests between controls and patients, run across all time points and in the theta frequency band (~4-7Hz) for the Fz electrode. The p-values of multiple point-by-point t-tests were corrected for false discovery rate (FDR) with utilizing the method described by Benjamini and Yekutieli [48]. The alpha was set to 5%, and results were deemed significant if the probability of type I error (false positives) was also lower than 5%. These corrected point-by-point exploratory comparisons indicated group differences in a window (~200-350 ms) in theta power (adjusted p<0.047 for all comparisons). There were no significant differences in PL values. Average power values from the significant time-frequency interval were then subjected to a two-sample t-test comparing the groups.

### Statistical Methods

IBM Statistical Package for Social Sciences (SPSS) 19.0 was used to analyse data. When comparing two groups, Student’s t test was used where data were normally distributed. Results were deemed significant in 95% Confidence Interval (CI). The majority of CANTAB test performance scores were non-normally distributed due to the heteroskedasticity in these variables. To overcome the heteroskedasticity, log transformations were applied and comparative analyses were run with the log transformed values (Table 3). For investigating the associations between measures Spearman’s rank correlations were used. 

**Table 3:** Mean MMN amplitude, latency, and power in patients and controls.

	Patients (n=19)	Controls (n=19)	Statistics (t or F values)	p value	95% Confidence Intervals
MMN peak amplitude (microvolts)	-0.9 (0.52)	-1.4 (0.4)	t = 3.22 df = 38	0.002	0.18 - 0.77
MMN peak latency (milliseconds)	166.8 (38)	167.2 (39)	t = -0.34 df = 38	0.70	-2.54 - 1.81
Standard peak amplitude	-0.37	-0.056	t = 1.16 df = 39	0.72	-0.23 - 0.85
Deviant peak amplitude	-1.02	-1.29	t = -0.81 df = 39	0.81	-0.92 - 0.39
Left Mastoid MMN peak amplitude	1.38629	1.82874	t = 1.94 df = 39	0.059	-0.02 - 0.9
Right Mastoid MMN peak amplitude	1.62725	1.97666	t = 1.31 df = 39	0.19	-0.19- 0.9
Theta band power (ERSP) (170-210ms)	0.12 (0.87)	0.71 (0.7)	t = -1.97 df = 38	0.06	-1.02 - -0.014

## Results

### MMN results

**Figure 1:**
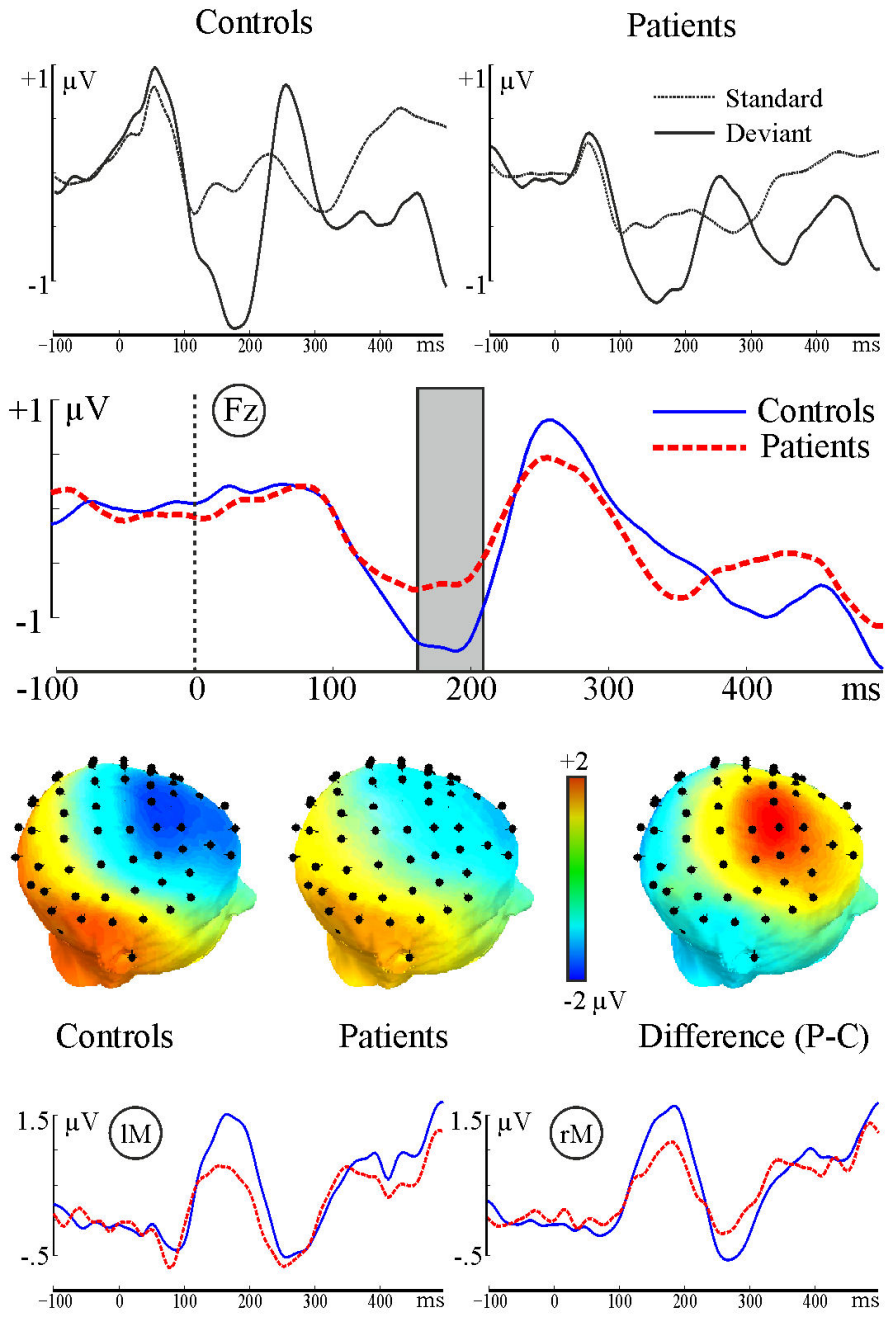
MMN waveforms and topographic headplots. First row: Standard and deviant waveforms from electrode Fz for Controls and Patients. Second row: MMN waveforms from electrode Fz for Controls and Patients. Significant differences were found in the marked (170-210ms) time interval. Third row: topographic headplots for two groups and their difference from the significant time interval (170-210ms). Electrodes with significant effects are marked by white disks. Fourth row: Difference waves from the mastoid electrodes.

The point-by-point analysis detected significant MMN amplitude differences between two groups between 170-210 ms post-stimulus over several fronto-central electrode sites (Figure 1). Mean amplitude of the MMN peak (170-210 ms) and MMN peak latency from the Fz electrode were then compared. MMN amplitude was significantly lower in patients with schizophrenia (t(38)=3.22, p=0.002, η=0.46). The MMN peak latency was not significantly different between groups (Table 3). 

The Group × Condition ANOVA comparing the mean amplitudes of the standard and deviant peaks within and across the two groups yielded a significant main effect of condition indicating that ERP amplitudes in the deviant condition (compared to the standard condition) were more negative in both groups (F(1,39)=123.2, p<0.0001). The main effect of group was not significant (F(1,39)=0.0069, p>0.9). The interaction of group and condition was significant (F(1,39)=11.28, p=0.002). Post-hoc group comparisons for the mean amplitudes of the standard and deviant peaks are reported in Table 3 (corrected p-values are presented). The pairwise comparison of standards and deviants did not yield significant differences (both p>0.7) indicating that the processing differences between patients and controls could not be explained by the type of the stimulus.

Comparison of the two groups yielded marginally significant group difference in the MMN amplitude at the left mastoid electrode site (t(39)=1.94, p=0.059) but not at the right mastoid electrode site (t(39)=1.31, p=0.19).

### Time-frequency power and phase-locking results

**Figure 2:**
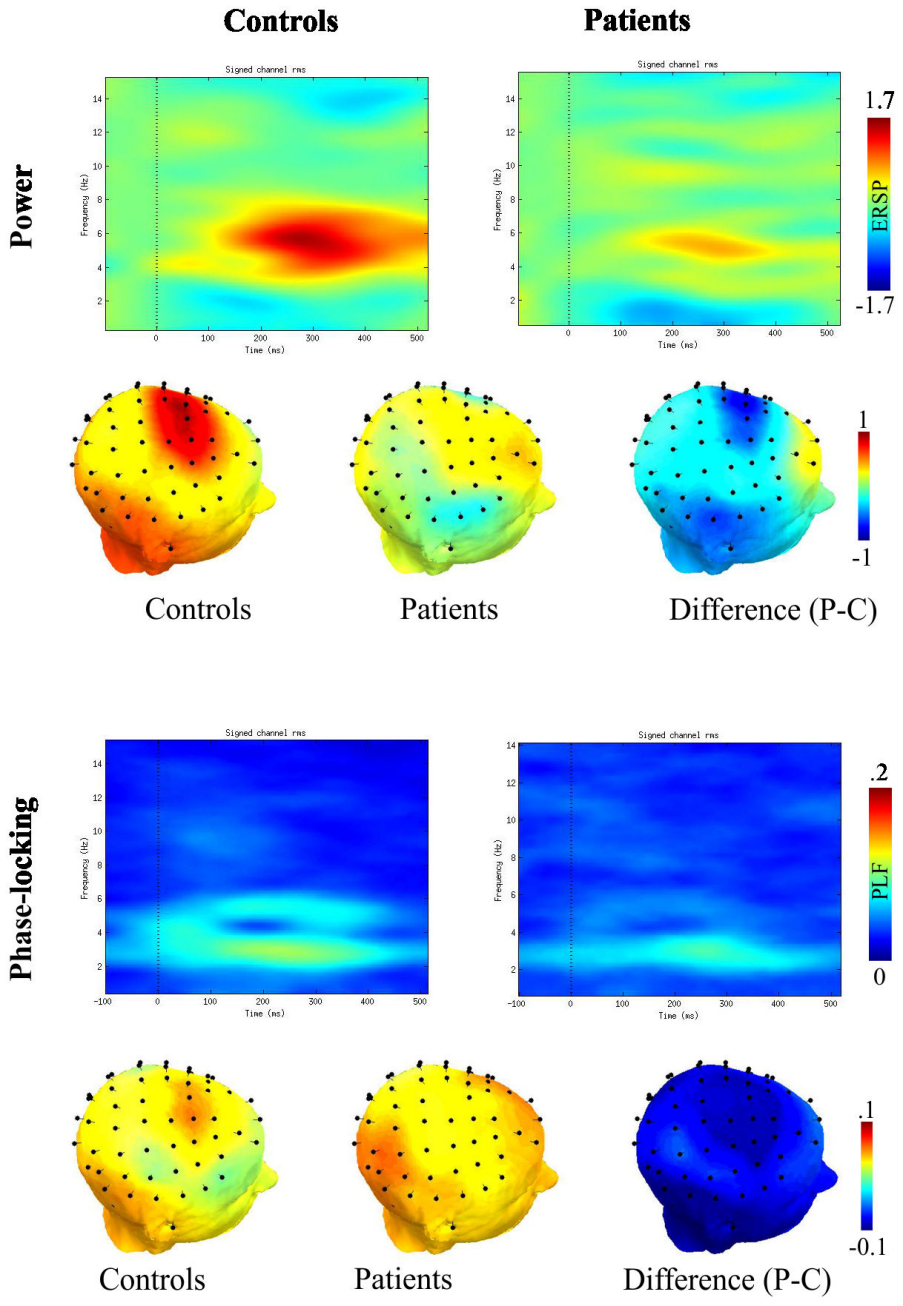
Power and Phase Locking Results for Controls and Patients with Schizophrenia. Upper panel: time-frequency power spectrum (ERSP) from the Fz electrode for Controls and Patients. The significant time-frequency segment overlapping in time with the MMN is marked with a black box in the figures (~3-6 Hz, around 200 ms). Lower panel: time-frequency phase-locking factor (PLF) for groups. Inserts in each panel show the topographic distribution of power and phase-locking values across all the electrodes.

A well-defined time-frequency interval within the time-frequency landscape showed significant group differences in power, but not in phase-locking (Figure 2). Control participants showed significantly larger trial-by-trial EEG power (t(38)=-1.97, p=0.056, see Table 3 and Figure 2; it is to note that the point-by-point corrected statistics were more sensitive to the effect, p-values <0.047 for all comparisons, for power group difference η=0.24). Correlation analysis among MMN amplitude and power was conducted in order to test whether theta oscillations in the time-frequency explain MMN amplitudes in the time domain. The correlation between theta power and MMN amplitude was significant (r=-0.34, p=0.032). After excluding two outliers (exceeding two standard deviations from the mean) the correlation was still significant (r=-0.35, p=0.03).

### Neurocognitive Performance

Patients with schizophrenia had poorer performance on the CANTAB tests of working memory, executive function, episodic memory and attention (Table 3). Social cognition performance was comparable between groups with the exception of the ‘surprise’ subdomain of the ERT, where patients performed poorer (Table 4). 

**Table 4:** Group comparisons of mean scores for CANTAB tests (log-transformed values).

	Patients (n=21)	Controls (n=21)	Statistics (t and df values)	95% Confidence Intervals	p value
SWM-errors 8 box	2.57 (1.40)	1.64 (1.82)	t = 1.842 df = 40	-0.92 - 1.94	0.07
SWM-errors 6 box	1.47 (1.19)	0.61 (0.92)	t = 2.611 df = 40	0.19 - 1.52	0.01
SWM-errors 4 box	0.44 (0.73)	0.05 (0.21)	t = 2.370 df = 40	0.55-0.74	0.02
SWM-strategy	14.48 (5.21)	13.52 (3.50)	t = 0.695 df = 40	-1.82 - 3.73	0.49
IED-EDS-errors	2.54 (1.95)	1.29 (0.84)	t = 2.625 df = 38	0.27 - 2.22	0.01
IED-total errors adjusted	5.79 (3.01)	3.50 (1.41)	t = 3.071 df = 38	0.75 - 3.81	0.005
OTS-mean choices-correction	1.26 (0.18)	1.10 (0.55)	t = 3.558 df = 37	0.06 - 0.24	0.002
OTS-mean latency-correction (ms)	9.60 (0.48)	9.65 (0.32)	t = -0.356 df = 37	-0.31 - 0.22	0.72
RVP-Sensitivity to Errors	0.87 (0.07)	0.95 (0.03)	t = -3.619 df = 33	-0.11 - -0.03	0.003
RVP-mean latency (ms)	6.02 (0.24)	5.90 (0.18)	t = 1.767 df = 33	-0.02 - 0.28	0.09
PAL-errors adjusted	5.58 (2.54)	2.07 (1.25)	t = 5.671 df = 40	2.24 - 4.77	<0.001
ERT-disgust (% correct)	1.26 (0.46)	1.46 (0.21)	t = -1.731 df = 39	-0.43 - 0.03	0.09
ERT-fear (% correct)	1.03 (0.42)	1.01 (0.37)	t = -0.100 df = 39	-0.24 - 0.26	0.92
ERT-happiness (% correct)	1.46 (0.35)	1.50 (0.21)	t = -0.497 df = 39	-0.23 - 0.13	0.61
ERT-sadness (% correct)	1.11 (0.43)	1.34 (0.32)	t = -1.920 df = 39	-0.47 - 0.01	0.06
ERT-surprise (% correct)	1.25 (0.38)	1.48 (0.18)	t = -2.429 df = 39	-0.42 - -0.03	0.02
ERT-anger (% correct)	1.14 (0.41)	1.24 (0.27)	t = -0.888 df = 39	-0.32 - 0.12	0.38
ERT-neutral (% correct)	1.25 (0.38)	1.44 (0.21)	t = -1.852 df = 39	-0.38 - 0.01	0.70

### Correlations between clinical, neurocognitive measures and MMN

No significant associations were detected between mean CANTAB performance and MMN amplitude or power in patients with schizophrenia (Table 5). The only exception was fear subdomain of ERT which correlated with MMN amplitude, although this significance disappeared when corrected for multiple comparisons. Furthermore none of the clinical measures, including functional outcome, was associated with any MMN measure (Table 6).

**Table 5:** Correlation coefficients between MMN measures and CANTAB performance (patients with schizophrenia).

	MMN amplitude	p value	MMN power	p value
SWM-errors 4 box	-0.1089	0.64	-0.014	0.96
SWM-errors 6 box	-0.0531	0.82	-0.2272	0.46
SWM-errors 8 box	0.2780	0.24	-0.1466	0.53
SWM-strategy	0.2818	0.24	0.0386	0.87
IED-EDS-errors	-0.1196	0.62	-0.2974	0.32
IED-total errors adjusted	-0.1644	0.50	-0.2606	0.39
OTS-mean choices-correction	0.0414	0.87	-0.0969	0.75
OTS-mean latency-correction (ms)	-0.3115	0.20	0.0299	0.90
RVP-Sensitivity to Errors	-0.1465	0.60	-0.0629	0.84
RVP-mean latency (ms)	0.2323	0.42	0.1500	0.59
PAL-errors adjusted	-0.0858	0.71	-0.2163	0.48
ERT-surprise (% correct)	0.2970	0.21	-0.0527	0.86
ERT-disgust (% correct)	-0.1128	0.65	0.2542	0.30
ERT-fear (% correct)	-0.5516	0.01	0.1188	0.62
ERT-happiness (% correct)	-0.4391	0.06	0.0313	0.89
ERT-sadness (% correct)	0.0145	0.95	0.2919	0.22
ERT-anger (% correct)	-0.2297	0.34	0.1687	0.48
ERT-neutral (% correct)	-0.0103	0.96	-0.1673	0.49

**Table 6:** Correlation coefficients between MMN measures and clinical variables (patients with schizophrenia).

	MMN amplitude	p value	MMN power	p value
Medication (mg) *	0.2638	0.54	-0.1724	0.52
Medication duration (years)	0.2297	0.39	-0.0524	0.84
BPRS	0.1996	0.45	0.3908	0.13
PANSS-General Psychopathology	0.0270	0.92	0.4978	0.050
PANSS-Negative	-0.1043	0.70	-0.1900	0.48
PANSS-Positive	0.1660	0.53	0.4938	0.052
BDI	-0.1930	0.47	0.2365	0.37
BAI	-0.2195	0.41	0.2933	0.27
NART	-0.3087	0.24	-0.0177	0.94
WASI	-0.2604	0.33	0.2489	0.35
WSAS	-0.3509	0.1410	-0.1813	0.45

^*^ Chlorpromazine equivalent dose

## Discussion

In this study the oscillatory activity underlying the MMN deficit in patients with schizophrenia was examined and we report for the first time, that the MMN deficits in patients with schizophrenia are associated with reduced theta power. While patients with schizophrenia showed significant impairments in multiple cognitive domains (with effect sizes ranging from 0.39 to 1.75, Table 4), these impairments were not associated with MMN amplitude, power or phase locking. Moreover, no significant association was detected between MMN measures and clinical parameters, including functional outcome. 

In the present study, duration MMN impairments in amplitude were observed in patients with schizophrenia (Figure 1). These impairments were of moderate effect size and were consistent with previous findings [10]. Reduced MMN amplitude observed in patients with schizophrenia suggests that their ability to discriminate sensorial differences is reduced [49]. This failure can result in impaired ability to predict and evaluate salience [49]. Further analysis of standard and deviant responses for each group suggested that the MMN amplitude differences between patients and controls were not driven by stimulus type. Instead, the significant interaction of group and stimulus type, and the significant group difference in the MMN (Deviant-Standard) amplitude, indicates that change detection was impaired in patients with schizophrenia, possibly as a result of the combination of responses to deviant and standard stimuli. However, it should be noted that MMN amplitude is regarded as the averaged end product of the underlying neuronal activity. Trial-by trial analysis of MMN in the time-frequency domain can provide a more sensitive measure of the underlying brain dynamics of MMN deficits in schizophrenia. 

**Figure 3:**
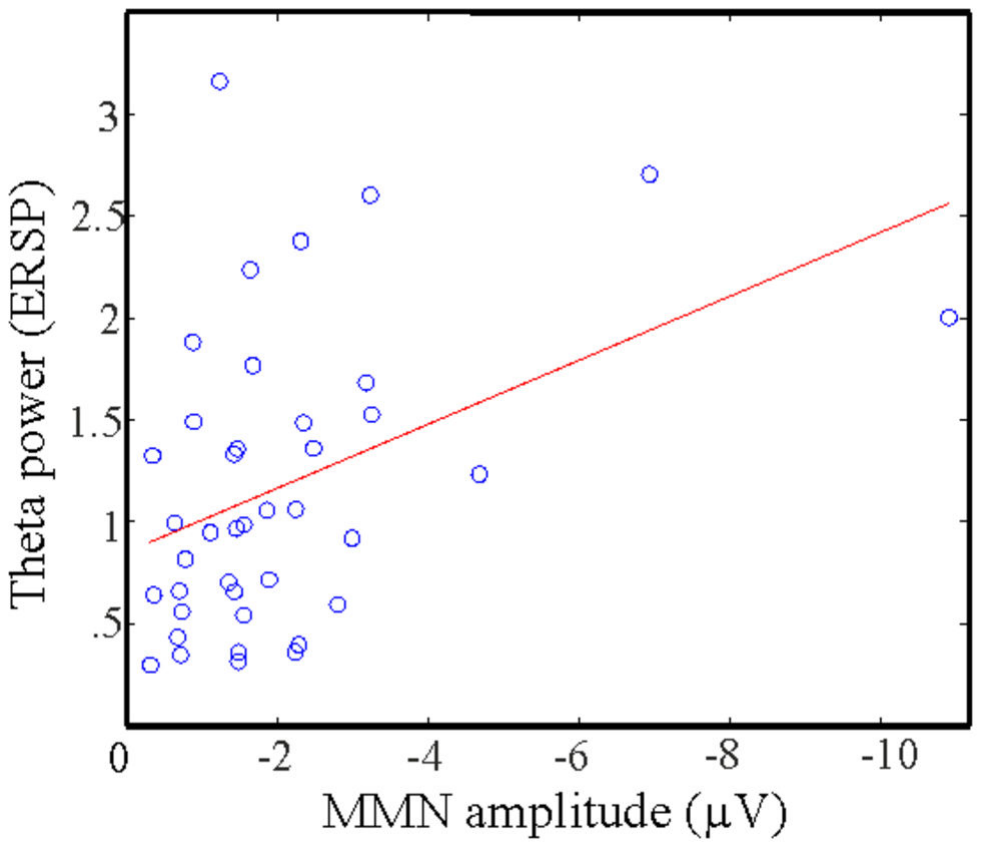
Correlation between MMN amplitude and theta power. The correlation remains significant after the removal of outliers (see Results).

Time-frequency analyses showed that patients with schizophrenia had reduced power in the theta band compared to controls (Figure 2, Table 3). Furthermore, the power of the theta band oscillation showed a significant correlation with the amplitude of the MMN. This significant correlation between theta power and time-domain MMN is consistent with previous studies indicating that MMN is associated with an increase in theta power in deviant trials [33,34]. A MEG study also demonstrated that theta power and phase coherence were increased in response to deviant stimulus around 150-200 ms [50]. Increased frontal theta activity has generally been associated with information processing and error monitoring [51-53]. Previous studies reported that patients with schizophrenia exhibited decreased delta and theta activity in response to novelty [31,32,54]. Findings from this study support the view that reduced theta power may be the underlying oscillatory mechanism related to the decreased MMN amplitudes in patients as reduced theta power was correlated with decreased MMN amplitude in schizophrenia patients (Figure 3). However, it is important to note here that due to the correlational nature of the MMN – theta power relationship, we cannot draw firm directional conclusions. Furthermore, the significant effects measured in theta power span across a wider time window (~200-350 ms) than the window of the peak MMN (170-210ms). Also, according to the corrected point-by-point statistics, the effects seen in the time-domain MMN arose somewhat earlier (170ms), than the onset of the effect seen in the time-frequency domain. Similar timing or window length differences between averaged time-domain MMN and trial-by-trial theta power could be observed earlier [34,50]. Therefore, beyond the fact that the two phenomena must, at least in part, reflect the same underlying neural mechanism, we cannot draw directional conclusions between theta power and time-domain MMN. 

While the spectral power is associated with the firing activity of stimulus-related neural networks, phase locking is related to the temporal synchronisation, or with the temporal re-setting of activity of these neuronal populations as response to the stimuli. Therefore phase locking can provide information about the synchrony of task related oscillatory activity which might have contributed to the MMN. However, in the present study phase locking was not significantly different between patients and controls, suggesting that at least in this population of patients, there is no abnormality in task related synchronization. However, these findings require confirmation in a larger study.

MMN impairments in amplitude and power were independent from symptom severity and antipsychotic medication dose. In the current study all patients were on atypical antipsychotics, and given the sample size it was not possible to examine the relationship between antipsychotic type/dose and MMN impairments. However, the results are consistent with the findings from a large study which indicated that MMN deficits were comparable among the patients who were on different atypical antipsychotics [55]. It should also be noted that there are studies showing positive effects of atypical antipsychotic medication on MMN [56]. Antidopaminergic antipsychotic drugs have a rather small effect on the course of cognitive impairment in schizophrenia [57]. On the other hand, dysfunctional glutamatergic transmission has been suggested to have more significant role in pathophysiology of cognitive impairments in schizophrenia [58]. Previous research indicated that modulation of the glutamatergic system (via NMDA receptor modulation) (and not dopamine or serotonin) was associated with MMN generation in auditory cortex [59,60]. Ketamine, an NMDA receptor antagonist was shown to reduce MMN amplitude in patients and healthy controls [13]. Also, NMDA receptor blockade was shown to reduce theta frequency power [61] and phase locking [62] in experimental studies. Glutamate (via NMDA receptors) might have a modulatory effect on neural populations within the cortical MMN generators (i.e. pyramidal cortical neurons, located in prefrontal and superior temporal cortices) which are also within the critical neural systems for pathophysiology of schizophrenia. In light of these findings, it can be suggested that MMN deficits in schizophrenia might be related to dysfunctional neural processing at prefrontal and temporal brain circuits, possibly linked to impaired glutamatergic neurotransmission. Furthermore, the findings should be evaluated in the context of possible biomarker properties of MMN. NMDA receptors and the glutamatergic system seem to be promising targets for novel drugs in schizophrenia [63,64]. In future studies, oscillatory activity measures underlying MMN may be used as the outcome measures for novel drug treatments. Preclinical models are already available in rodents [65] and macaque [58] and NMDA antagonist MK-801 induced MMN deficits have been shown [14]. Thus, drug discovery research might investigate neurotransmitter systems associated with MMN, and MMN can be used as a candidate biomarker to test new drugs in animal psychosis models (back-translation). 

In this sample of patients, we observed no relationship between MMN and clinical, functional or cognitive outcome measures. Previous studies investigating the association between neurocognitive impairments and MMN amplitude deficits in schizophrenia have yielded inconsistent results. Some studies reported significant correlations between MMN and specific cognitive domains [16,24] while some failed to detect correlations [25]. In the current study, the relationship between oscillatory activity and cognitive function was examined for the first time in addition to the standard MMN amplitude, and these findings consistently failed to identify a relationship with cognitive function. Findings from the present study suggested that MMN might not be a sensitive marker for cognitive impairment in schizophrenia. However, there are only a few studies, including the current one, that have examined the relationship between MMN and cognition and more data from larger studies are needed to comment on the associations between cognitive impairments at the neuropsychological level and MMN deficits. Schizophrenia is a chronic, debilitating condition and biomarkers that can allow predictions about the daily functioning of patients are notably important. Previously, the association between the MMN amplitude and work functioning have been shown [17,66-68]. However, the functional outcome scores of the patients were not correlated with any of the MMN measures in this study. The disparity might be related to the different outcome measures used (GAF Vs. WSAS) or the sample size. Indeed studies reporting association between functional outcome and MMN deficits had larger samples [17,66-68], so it is possible that non-replication in our study might be driven by the small sample size. 

A few methodological considerations should be listed with regards to this study. First, the study included only patients with a chronic history of schizophrenia and all the patients were taking atypical antipsychotic drugs. The findings needs to be replicated with patient samples including first episode patients and drug-naïve patients as well as patients taking typical antipsychotic drugs. Secondly, we used a roving duration MMN paradigm and while the impairments in MMN were comparable to standard MMN paradigms, the lack of relationship observed with cognition in this study may be specific to the roving paradigm and need to be confirmed with other MMN paradigms. 

In summary, the present study showed for the first time that MMN impairments in schizophrenia were linked to reduced theta power but not phase synchrony. It also showed that neither MMN amplitude or theta power was related to impairments in clinical, functional or cognitive outcome measures. These findings suggest that while MMN may be a useful marker to probe NMDA receptor mediated mechanisms and associated impairments in gain control and perceptual changes, it may not be a useful marker in association with clinical or cognitive changes. Further studies addressing the underlying oscillatory mechanisms of MMN with different paradigms and with possible pharmacological interventions might have implications for the use of MMN as a biomarker in drug discovery.

## Supporting Information

Information S1 **Consent form: Participant consent form for the study.**
(PDF)

Information S2 **Information Leaflet for patients: Information leaflet about the study tailored for patients.**
(PDF)

Information S3 **Information Leaflet for healthy volunteers: Information leaflet about the study for healthy volunteers.**
(PDF)

## References

[1] Kurtz MM (2005) Neurocognitive impairment across the lifespan in schizophrenia: an update. Schizophr Res 74(1): 15–26. doi:10.1016/j.schres.2004.07.005. PubMed: 15694750. 15694750

[2] Fioravanti M, Carlone O, Vitale B, Cinti ME, Clare L (2005) A meta-analysis of cognitive deficits in adults with a diagnosis of schizophrenia. Neuropsychol Rev 15(2): 73–95. doi:10.1007/s11065-005-6254-9. PubMed: 16211467. 16211467

[3] Kurtz MM, Tolman A (2011) Neurocognition, Insight into Illness and Subjective Quality-of-Life in Schizophrenia: What is Their Relationship? Schizophr Res 127(1-3): 157-162. doi:10.1016/j.schres.2010.12.004. PubMed: 21211943. 21211943 PMC3051009

[4] Butler PD, Chen Y, Ford JM, Geyer MA, Silverstein SM et al. (2012) Perceptual Measurement in Schizophrenia: Promising Electrophysiology and Neuroimaging Paradigms From CNTRICS. Schizophr Bull 38(1): 81-91. doi:10.1093/schbul/sbr106. PubMed: 21890745. 21890745 PMC3245585

[5] Näätänen R, Gaillard AWK, Mäntysalo S (1978) Early selective-attention effect on evoked potential reinterpreted. Acta Psychol (Amst) 42: 313–329. doi:10.1016/0001-6918(78)90006-9. PubMed: 685709. 685709

[6] Todd J, Michie PT, Schall U, Ward PB, Catts SV (2012) Mismatch negativity (MMN) reduction in schizophrenia-impaired prediction-error generation, estimation or salience? International Journal of Psychophysiology 83(2): 222–231. doi:10.1016/j.ijpsycho.2011.10.003. PubMed: 22020271. 22020271

[7] Shelley AM, Ward PB, Catts SV, Michie PT, Andrews S et al. (1991) Mismatch negativity: an index of a preattentive processing deficit in schizophrenia. Biol Psychiatry 30: 1059-1062. doi:10.1016/0006-3223(91)90126-7. PubMed: 1756198. 1756198

[8] Devrim-Uçok M, Keskin-Ergen HY, Uçok A (2008) Mismatch negativity at acute and post-acute phases of first-episode schizophrenia. Eur Arch Psychiatry Clin Neurosci 258(3): 179–185. doi:10.1007/s00406-007-0772-9. PubMed: 18000635. 18000635

[9] Umbricht D, Koller R, Schmid L, Skrabo A, Grübel C et al. (2003) How Specific Are Deficits in Mismatch Negativity Generation to Schizophrenia? Biol Psychiatry 53: 1120-1131. doi:10.1016/S0006-3223(02)01642-6. PubMed: 12814863. 12814863

[10] Umbricht D, Krljes S (2005) Mismatch negativity in schizophrenia: A meta-analysis. Schizophr Res 76: 1–23. doi:10.1016/j.schres.2004.12.002. PubMed: 15927795. 15927795

[11] Korostenskaja M, Dapsys K, Siurkute A, Maciulis V, Ruksenas O et al. (2005) Effects of olanzapine on auditory P300 and mismatch negativity (MMN) in schizophrenia spectrum disorders. Prog Neuropsychopharmacol Biol Psychiatry 29(4): 543–548. doi:10.1016/j.pnpbp.2005.01.019. PubMed: 15866356. 15866356

[12] Schall U, Catts SV, Chaturvedi S, Liebert B, Redenbach J et al. (1998) The effect of clozapine therapy on frontal lobe dysfunction in schizophrenia: neuropsychology and event-related potential measures. Int J Neuropsychopharmacol 1(1): 19–29. doi:10.1017/S146114579800100X. PubMed: 11281941. 11281941

[13] Krystal JH, Karper LP, Seibyl JP, Freeman GK, Delaney R et al. (1994) Subanesthetic Effects of the Noncompetitive NMDA Antagonist, Ketamine in Humans: Psychotomimetic, Perceptual, Cognitive, and Neuroendocrine Responses. Arch Gen Psychiatry 51(3): 199-214. doi:10.1001/archpsyc.1994.03950030035004. PubMed: 8122957. 8122957

[14] Tikhonravov D, Neuvonen T, Pertovaara A, Savioja K, Ruusuvirta T et al. (2008) Effects of an NMDA-receptor antagonist MK-801 on an MMN-like response recorded in anesthetized rats. Brain Res 1203(8): 97–102. PubMed: 18325485. 18325485 10.1016/j.brainres.2008.02.006

[15] Cooper RJ, Atkinson RJ, Clark RA, Michie PT (2013) Event-related potentials reveal modelling of auditory repetition in the brain. Int J Psychophysiol 88: 74-81. doi:10.1016/j.ijpsycho.2013.02.003. PubMed: 23454030. 23454030

[16] Baldeweg T, Klugman A, Gruzelier J, Hirsch SR (2004) Mismatch negativity potentials and cognitive impairment in schizophrenia. Schizophr Res 69: 203–217. doi:10.1016/j.schres.2003.09.009. PubMed: 15469194. 15469194

[17] Jahshan C, Cadenhead KS, Rissling AJ, Kirihara K, Braff DL et al. (2012) Automatic Sensory Information Processing Abnormalities across the Illness Course of Schizophrenia. Psychol Med 42(1): 85–97. doi:10.1017/S0033291711001061. PubMed: 21740622. 21740622 PMC3193558

[18] Light GA, Braff DL (2005) Stability of mismatch negativity deficits and their relationship to functional impairments in chronic schizophrenia. Am J Psychiatry 162: 1741–1743. doi:10.1176/appi.ajp.162.9.1741. PubMed: 16135637. 16135637

[19] Light GA, Swerdlow NR, Rissling AJ, Radant A, Sugar CA et al. (2012) Characterization of neurophysiologic and neurocognitive biomarkers for use in genomic and clinical outcome studies of schizophrenia. PLOS ONE 7(7): e39434. doi:10.1371/journal.pone.0039434. PubMed: 22802938. 22802938 PMC3389010

[20] Tervaniemi M, Lehtokoski A, Sinkkonen J, Virtanen J, Ilmoniemi RJ et al. (1999) Test-retest reliability of mismatch negativity for duration, frequency and intensity changes. Clin Neurophysiol 110: 1388-1393. doi:10.1016/S1388-2457(99)00108-X. PubMed: 10454274. 10454274

[21] Michie PT, Innes-Brown H, Todd J (2002) Duration mismatch negativity in biological relatives of patients with schizophrenia spectrum disorders. Biol Psychiatry 52(7): 749–758. doi:10.1016/S0006-3223(02)01379-3. PubMed: 12372666. 12372666

[22] Şevik AE, Anıl Yağcıoğlu E, Yağcıoğlu S, Karahan S, Gürses N et al. (2011) Neuropsychological performance and auditory event related potentials in schizophrenia patients and their siblings: a family study. Schizophr Res 130(1-3): 195–202. doi:10.1016/j.schres.2011.04.018. PubMed: 21592733. 21592733

[23] Bramon E, Croft RJ, McDonald C, Virdi GK, Gruzelier JG et al. (2004) Mismatch negativity in schizophrenia: a family study. Schizophr Res 67(1): 1-10. doi:10.1016/S0920-9964(03)00132-4. PubMed: 14741319. 14741319

[24] Toyomaki A, Kusumi I, Matsuyama T, Kako Y, Ito K et al. (2008) Tone duration mismatch negativity deficits predict impairment of executive function in schizophrenia. Progress in Neuropsychopharmacol and Biological Psychiatry 32(1): 95–99. 10.1016/j.pnpbp.2007.07.020 17764800

[25] Brockhaus-Dumke A, Tendolkar I, Pukrop R, Schultze-Lutter F, Klosterkötter J et al. (2005) Impaired mismatch negativity generation in prodromal subjects and patients with schizophrenia. Schizophr Res 73: 297–310. doi:10.1016/j.schres.2004.05.016. PubMed: 15653275. 15653275

[26] Levaux MN, Potvin S, Sepehry AA, Sablier J, Mendrek A et al. (2007) Computerized assessment of cognition in schizophrenia: Promises and pitfalls of CANTAB. Eur Psychiatry 22: 104-115. doi:10.1016/j.eurpsy.2007.01.330. PubMed: 17227707. 17227707

[27] Barnett JH, Robbins TW, Leeson VC, Sahakian BJ, Joyce EM et al. (2010) Assessing cognitive functions in clinical trials of schizophrenia. Neuroscience and Biobehav Reviews 34: 1161-1177. 10.1016/j.neubiorev.2010.01.012 20105440

[28] Robbins TW (2005) Synthesizing schizophrenia: a bottom-up, symptomatic approach. Schizophr Bull 31: 854-864. doi:10.1093/schbul/sbi044. PubMed: 16107585. 16107585

[29] Buzsáki G, Draguhn A (2004) Neuronal oscillations in cortical networks. Science 304(5679): 1926-1929. doi:10.1126/science.1099745. PubMed: 15218136. 15218136

[30] Başar E, Başar-Eroglu C, Karakaş S, Schürmann M (2001) Gamma, alpha, delta, and theta oscillations govern cognitive processes. International Journal of Psychophysiology 39(2): 241-248. 11163901 10.1016/s0167-8760(00)00145-8

[31] Kirino E (2007) Mismatch negativity correlates with delta and theta EEG power in schizophrenia. Int J Neurosci 17(9): 1257-1279. PubMed: 17654091. 10.1080/00207450600936635 17654091

[32] Rodionov V, Durst R, Mager M, Teitelbaum A, Raskin S et al. (2009) Wavelet Analysis of the Frontal Auditory Evoked Potentials Obtained in the Passive Oddball Paradigm in Healthy Subjects and Schizophrenics. J Basic Clin Physiol Pharmacol 20(3): 233-264. PubMed: 19852310. 19852310 10.1515/jbcpp.2009.20.3.233

[33] Fuentemilla L, Marco-Pallarés J, Münte TF, Grau C (2008) Theta EEG oscillatory activity and auditory change detection. Brain Res 1220: 93-101. doi:10.1016/j.brainres.2007.07.079. PubMed: 18076870. 18076870

[34] Ko D, Kwon S, Lee GT, Im CH, Kim KH et al. (2012) Theta Oscillation Related to the Auditory Discrimination Process in Mismatch Negativity: Oddball versus Control Paradigm. J Clin Neurol 8(1): 35-42. doi:10.3988/jcn.2012.8.1.35. PubMed: 22523511. 22523511 PMC3325430

[35] Makeig S (1993) Auditory event-related dynamics of the EEG spectrum and effects of exposure to tones. Electroencephalogr Clin Neurophysiol 86(4): 283-293. doi:10.1016/0013-4694(93)90110-H. PubMed: 7682932. 7682932

[36] Bishop DVM, Hardiman MJ (2010) Measurement of mismatch negativity in individuals: A study using single trial analysis. Psychophysiology 47(4): 697-705. PubMed: 20210877. 20210877 10.1111/j.1469-8986.2009.00970.x PMC2904495

[37] Delorme A, Makeig S (2004) EEGLAB: an open source toolbox for analysis of single-trial EEG dynamics including independent component analysis. J Neurosci Methods 134(1): 9-21. doi:10.1016/j.jneumeth.2003.10.009. PubMed: 15102499. 15102499

[38] Tallon-Baudry C, Bertrand O, Delpuech C, Pernier J (2004) Stimulus specificity of phase-locked and non-phase-locked 40 Hz visual responses in human. Journal of Neuroscience 16(13): 4240-4249. PubMed: 8753885. 10.1523/JNEUROSCI.16-13-04240.1996 PMC6579008 8753885

[39] Sheehan DV, Lecrubier Y, Harnett-Sheehan K, Janavs J, Weiller E et al. (1997) Reliability and Validity of the MINI International Neuropsychiatric Interview (M.I.N.I.) according to the SCID-P. European Psychiatry 12: 232-241. doi:10.1016/S0924-9338(97)80737-7.

[40] Kay SR, Fiszbein A, Opler LA (1987) The positive and negative syndrome scale for schizophrenia. Schizophr Bull 13: 261–276. doi:10.1093/schbul/13.2.261. PubMed: 3616518. 3616518

[41] Flemenbaum A, Zimmermann RL (1973) Inter and intra-rater reliability of the Brief Psychiatric Rating Scale. Psychol Rep 32(3): 783-792. PubMed: 4704758. 4704758 10.2466/pr0.1973.33.3.783

[42] Beck AT, Steer RA, Brown GK (1996) BDI-II: Beck Depression Inventory-II. Psychological Corp, San Antonio, TX..

[43] Steer RA, Rissmiller DJ, Ranieri WF, Beck AT (1993) Structure of the computer-assisted Beck Anxiety Inventory with psychiatric inpatients. J Pers Assess 60(3): 532-542. doi:10.1207/s15327752jpa6003_10. PubMed: 8336268. 8336268

[44] Axelrod BN (2002) Validity of the Wechsler abbreviated scale of intelligence and other very short forms of estimating intellectual functioning. Assessment 9: 17–23. doi:10.1177/1073191102009001003. PubMed: 11911230. 11911230

[45] Bright P, Jaldow E, Kopelman MD (2002) The National Adult Reading Test as a measure of premorbid intelligence: a comparison with estimates derived from demographic variables. J Int Neuropsychol Soc 8(6): 847-854. doi:10.1017/S1355617702860131. PubMed: 12240749. 12240749

[46] Mundt JC, Marks IM, Shear KM, Greist JH (2002) The Work and Social Adjustment Scale: a simple measure of impairment in functioning. Br J Psychiatry 180(5): 461–464. doi:10.1192/bjp.180.5.461. PubMed: 11983645. 11983645

[47] CANTABeclipse, 2011 Test Administration Guide. Cambridge Cognition Limited Manual Version 4.0.0

[48] Benjamini Y, Yekutieli D (2001) The control of the false discovery rate in multiple testing under dependency. Annals of Statistics: 1165-1188.

[49] Friston K (2005) A theory of cortical responses. Philosophical transactions of the Royal Society B: Biological sciences 360 (1456): 815–36 10.1098/rstb.2005.1622 PMC1569488 15937014

[50] Hsiao FJ, Wu ZA, Ho LT, Lin YY (2009) Theta oscillation during auditory change detection: an MEG study. Biol Psychol 81(1): 58-66. doi:10.1016/j.biopsycho.2009.01.007. PubMed: 19428969. 19428969

[51] Başar-Eroglu C, Başar E, Demiralp T, Schürmann M (1992) P300-response: possible psychophysiological correlates in delta and theta frequency channels. A review. Int J Psychophysiol 13(2): 161-179. doi:10.1016/0167-8760(92)90055-G. PubMed: 1399755. 1399755

[52] Yordanova J, Falkenstein M, Hohnsbein J, Kolev V (2004) Parallel systems of error processing in the brain. NeuroImage 22(2): 590-602. doi:10.1016/j.neuroimage.2004.01.040. PubMed: 15193587. 15193587

[53] Demiralp T, Ademoglu A, Comerchero M, Polich J (2001) Wavelet analysis of P3a and P3b. Brain Topogr 13(4): 251-267. doi:10.1023/A:1011102628306. PubMed: 11545154. 11545154

[54] Bates AT, Kiehl KA, Laurens KR, Liddle PF (2009) Low-frequency EEG oscillations associated with information processing in schizophrenia. Schizophr Res 115(2): 222-230. PubMed: 19850450. 19850450 10.1016/j.schres.2009.09.036

[55] Rissling AJ, Braff DL, Swerdlow NR, Hellemann G, Rassovsky Y et al. (2012) Disentangling early sensory information processing deficits in schizophrenia. Clin Neurophysiol 123: 1942–1949. doi:10.1016/j.clinph.2012.02.079. PubMed: 22608970. 22608970 PMC3436955

[56] Zhou Z, Zhu H, Chen L (2013) Effect of Aripiprazole on Mismatch Negativity (MMN) in Schizophrenia. PLOS ONE 8(1): e52186. doi:10.1371/journal.pone.0052186. PubMed: 23308105. 23308105 PMC3538635

[57] Keefe RE, Bilder RM, Davis SM, Harvey PD, Palmer BW et al. (2007) Neurocognitive Effects of Antipsychotic Medications in Patients with Chronic Schizophrenia in the CATIE Trial. Arch Gen Psychiatry 64(6): 633-647. doi:10.1001/archpsyc.64.6.633. PubMed: 17548746. 17548746

[58] Coyle JT (2006) Glutamate and schizophrenia: beyond the dopamine hypothesis. Cell Mol Neurobiol 26: 365–384. PubMed: 16773445. 16773445 10.1007/s10571-006-9062-8 PMC11881825

[59] Javitt DC, Steinschneider M, Schroeder CE, Arezzo JC (1996) Role of cortical N-methyl-D-aspartate receptors in auditory sensory memory and mismatch negativity generation: implications for schizophrenia. Proc Natl Acad Sci U S A 93(21): 11962–11967. doi:10.1073/pnas.93.21.11962. PubMed: 8876245. 8876245 PMC38166

[60] Leung S, Croft RJ, Guille V, Scholes K, O’Neill BV et al. (2010) Acute dopamine and/or serotonin depletion does not modulate mismatch negativity (MMN) in healthy human participants. Psychopharmacology (Berl) 208(2): 233-244. doi:10.1007/s00213-009-1723-0. PubMed: 20012022. 20012022

[61] Lazarewicz MT, Ehrlichman RS, Maxwell CR, Gandal MJ, Finkel LH et al. (2010) Ketamine modulates theta and gamma oscillations. J Cogn Neurosci 22(7): 1452-1464. doi:10.1162/jocn.2009.21305. PubMed: 19583475. 19583475

[62] van Wingerden M, Vinck M, Tijms V, Ferreira IRS, Jonker AJ et al. (2012) NMDA Receptors Control Cue-Outcome Selectivity and Plasticity of Orbitofrontal Firing Patterns during Associative Stimulus-Reward Learning. Neuron 76(4): 813-825. doi:10.1016/j.neuron.2012.09.039. PubMed: 23177965. 23177965

[63] Buchanan RW, Freedman R, Javitt DC, Abi-Dargham A, Lieberman JA (2007) Recent advances in the development of novel pharmacological agents for the treatment of cognitive impairments in schizophrenia. Schizophr Bull 33(5): 1120–1130. doi:10.1093/schbul/sbm083. PubMed: 17641146. 17641146 PMC2632365

[64] Schmidt A, Bachmann R, Kometer M, Csomor PA, Stephan KE et al. (2012) Mismatch negativity encoding of prediction errors predicts S-ketamine-induced cognitive impairments. Neuropsychopharmacology 37(4): 865–875. doi:10.1038/npp.2011.261. PubMed: 22030715. 22030715 PMC3280661

[65] Astikainen P, Stefanics G, Nokia M, Lipponen A, Cong F et al. (2011) Memory-based mismatch response to frequency changes in rats. PLOS ONE 6(9): e24208. doi:10.1371/journal.pone.0024208. PubMed: 21915297. 21915297 PMC3167833

[66] Kawakubo Y, Kasai K (2006) Support for an association between mismatch negativity and social functioning in schizophrenia. Prog Neuropsychopharmacol Biol Psychiatry 30(7): 1367-1368. doi:10.1016/j.pnpbp.2006.03.003. PubMed: 16603302. 16603302

[67] Rasser PE, Schall U, Todd J, Michie PT, Ward PB et al. (2011) Gray matter deficits, mismatch negativity, and outcomes in schizophrenia. Schizophr Bull 37(1): 131-140. doi:10.1093/schbul/sbp060. PubMed: 19561058. 19561058 PMC3004193

[68] Wynn JK, Sugar C, Horan WP, Kern R, Green MF (2010) Mismatch Negativity, Social Cognition, and Functioning in Schizophrenia Patients. Biol Psychiatry 67(10): 940–947. doi:10.1016/j.biopsych.2009.11.024. PubMed: 20074704. 20074704 PMC2862843

