# Supplementary material for: Oscillatory Underpinnings of Mismatch Negativity and Their Relationship with Cognitive Function in Patients with Schizophrenia
Source: PLoS One. 2013 Dec 17;8(12):e83255. doi: 10.1371/journal.pone.0083255 (PMC3866183; doi:10.1371/journal.pone.0083255)
Supplement: Information S1 — Consent form: Participant consent form for the study. (PDF) [file pone.0083255.s001.pdf]

GlaxoSmithKline Research & Development Limited
Clinical Unit Cambridge, ACCI, Addenbrooke's Hospital, Cambridge.
Tel. 01223-296001

Study Number: EMI 111781
Volunteer Panel ID Number:
Investigator: Dr. Pradeep Nathan

PARTICIPANT CONSENT FORM

		Please initial each box
1	**I confirm that I have read and understand the participant information sheet version dated 26 August 2010 for the above study. I have had the opportunity to consider the information, ask questions and have had these answered satisfactorily.**	☐
2	**I understand that my participation is voluntary and that I am free to withdraw at any time, without giving any reason, without my medical care or legal rights being affected. I understand that data collected up to my time of withdrawal may be used. All data will be handled, stored and destroyed in compliance with the Data Protection Act (DPA).**	☐
3	**I understand that sections of my medical notes that are relevant to my taking part in this research may be looked at by responsible individuals from or delegated by GlaxoSmithKline, or from regulatory authorities. I have been assured that all data relating to my person will be treated with absolute confidentiality at all times and will not be made public. I give permission for these individuals to have access to my records.**	☐
4	**I agree that the Unit/Study Physicians may contact my General Practitioner to make known my participation in this study and I authorise my General Practitioner to disclose details of any relevant medical or drug history in confidence. In addition, I agree that my GP be informed of any unexpected result.**	☐
5	**I have read and understand the compensation arrangements for this study as specified in the information sheet.**	☐
6	**I have read, understand and agree to the study restrictions as specified in the information sheet.**	☐
8	**It has been explained to me that the procedures being tested in this study may involve risks to me which are currently unforeseeable.**	☐
9	**I agree to take part in the above study.**	☐

*** Delete as appropriate.**

**Name of volunteer**	**Signature**	**Date (DD/MMM/YYYY) Time (hh:mm)**
**Name of person taking consent (if not Principal Investigator)**	**Signature**	**Date (DD/MMM/YYYY) Time (hh:mm)**
**Principal Investigator**	**Signature**	**Date (DD/MMM/YYYY)**
