# Supplementary material for: Oscillatory Underpinnings of Mismatch Negativity and Their Relationship with Cognitive Function in Patients with Schizophrenia
Source: PLoS One. 2013 Dec 17;8(12):e83255. doi: 10.1371/journal.pone.0083255 (PMC3866183; doi:10.1371/journal.pone.0083255)
Supplement: Information S2 — Information Leaflet for patients: Information leaflet about the study tailored for patients. (PDF) [file pone.0083255.s002.pdf]

PARTICIPANT INFORMATION SHEET

(PATIENTS)

Study Identification: EMI 111781

Sponsor Company Name: GlaxoSmithKline Research & Development Ltd

Subject Identification Number: ....

1. STUDY TITLE

Structural and Functional Imaging & Cognitive Endophenotypes of Schizophrenia and their Longitudinal Variability

Short Title: Imaging and Cognitive Endophenotypes in schizophrenia

2. INVITATION PARAGRAPH

You have been asked to take part in a clinical research study but before you decide whether to take part it is important for you to know why the research is being done and what it involves. It is important that you read and understand the information given to you in this information sheet. Please ask any questions that you may have to the Principal Investigator’s team in the Clinical Unit Cambridge at the time of the consent talk or the subsequent pre-study medical examination. If you are satisfied with the information about the study and wish to take part in this study, you will be asked to sign the consent form; the doctor who discussed the study with you will also sign it.

- Section 1 tells you the purpose of this study and what will happen to you if you take part.
- Section 2 gives you more detailed information about the conduct of the study.

Please take time to read the following information carefully and discuss it if you wish with friends, relatives or your personal doctor (i.e., general practitioner). Ask any member of the research study if there is anything that is not clear or if you would like more information. Take time to decide whether or not you wish to take part.

Section 1: Purpose of the study and what will happen

3. WHAT IS THE PURPOSE OF THE STUDY?

The purpose of this study is to find out more about cognitive changes in schizophrenia using behavioural testing (i.e. testing of attention, learning and memory) and brain scans (while performing other attention and memory tasks), by comparing results of schizophrenia or schizoaffective disorder patients and healthy controls.

This form is intended to give you information about the procedures that will be performed.

4. WHY HAVE I BEEN INVITED?

You are being invited because you have a diagnosis of Schizophrenia or schizoaffective disorder and your doctor has informed you about this study.

5. DO I HAVE TO TAKE PART?

It is up to you to decide if you want to take part in this study. If you decide to take part, you will be asked to sign a consent form. If you sign the consent form you are still free to stop participating from the study at any time and do not have to give a reason. Withdrawing from the study will not affect the standard of care you receive.

6. WHAT WILL HAPPEN TO ME IF I DO TAKE PART?

There will be 3-4 visits, all performed on an outpatient basis approximately 1 week apart. The entire duration of the study will be three-four weeks. (Details of the procedures are explained in Q10)

On your screening visit, you will undergo screening to ensure you fit the eligibility criteria of the study. This includes a medical history and examination, urine drugs test, urine pregnancy test for females, alcohol breath test, blood pressure and suitability to have a magnetoencephalography (MEG) scan.

On the screening day, depending on MRI scanner availability, you may undergo an MRI scan and tests of attention, learning and memory (CANTAB). If not you will be asked to come back for Visit 1 which will consist of an MRI scan and tests of attention, learning and memory (CANTAB). On either screening day or visit 1 you will also be asked to fill in some questionnaires that assess your IQ, schizophrenia symptoms, depression and anxiety symptoms and other symptoms related to mental illness. You will also complete a questionnaire asking questions related to your work, home management, social leisure, private leisure, and relationships. Some of these questionnaires may be completed later, on visits 2 or 3.

Visits 2 and 3 will be identical study visits and will involve a urine drugs test, a pregnancy test for females, an alcohol breath test, a MEG scan, an EEG scan and tests of attention learning and memory (known as CANTAB).

Within a week after the last visit, you will receive a telephone call to ensure you are well.

The MRI scan will take place at the MRIS at Addenbrooke's Hospital. The MEG scan will be conducted at the MRC Cognition and Brain Sciences Unit in Cambridge. All other procedures will be conducted at the Clinical Unit Cambridge, Addenbrooke's Hopital.

Proposed individual schedule

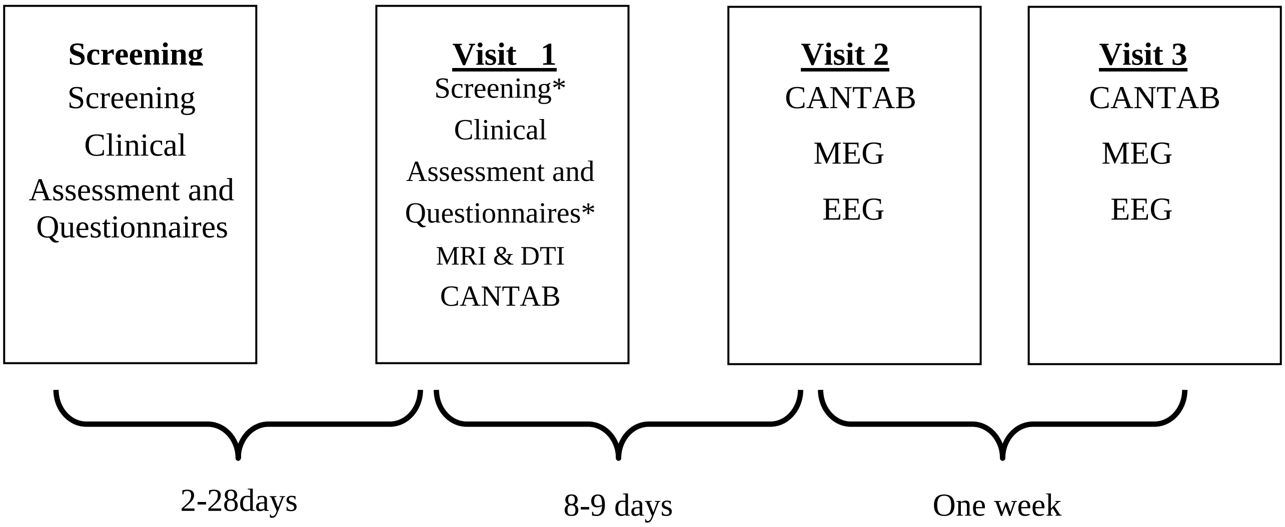

* Depending on MRI scanner availability screening and clinical assessment may be completed on the same day. In situations where this is not possible, participants will be screened separately and brought back approx 2-28 days later for visit 1 which will consist of MRI&DTI and CANTAB. Accordingly some participants may have 3 visits whereas some may have 4.

Duration of tests

MINI questionnaire	60 min
PANNS questionnaire	40 min
BPRS questionnaire	30 min
Beck Anxiety questionnaire	10 min
Beck Depression questionnaire	5 min
Wescheler abbreviated scale of	30 min

intelligence	
Work and Social Adjustment scale	5 min
National adult reading test (NART)	5-10 min
Handedness Inventory	1-3 min
MRI brain	40 min
CANTAB	60 min
EEG	60 min

7. WHAT ELSE WOULD I NEED TO DO IF I TAKE PART?

You are required to comply with the following instructions and restrictions:

Adverse Events	You should report any adverse events to the study staff. An adverse event is when you feel unwell or different in any way. After you have left the unit, you need to tell the study staff of any ongoing or new adverse events arising from the study.
Recreational drugs	You should not take recreational drugs within 48 hours of the study visits. Drugs tests will be done throughout the study. If a test is positive you will may be excluded from the study.
Smoking	If you are a smoker, you may smoke as normal except when on the Addenbrooke's campus where smoking is not allowed.
Alcohol	You must refrain from alcohol for at least 24 hours before the screening visit, and each visit to the unit and whilst in the unit. Men should drink no more than 21 units/week and women be restricted to 14 units/week. (1 unit is equivalent to a half-pint (220mL) of beer or 1 measure (25mL) of spirits or 1 glass (125mL) of wine).
Contraception	Females of child-bearing potential are allowed to participate in this study as long as they practise effective contraception from the Screening Visit to the last assessment visit, Visit 3.<br>The methods of contraception include:<br>o Complete abstinence<br>o Hormonal contraception<br>o Sterilization of subject's male partner prior to female subject's entry into study<br>o Any intra-uterine device with a documented failure rate of less than 1% per year<br>o Double barrier method if comprised of condom or occlusive cap (diaphragm or cervical/vault caps) plus spermicidal agent (foam/gel/film/cream/suppository)
Exercise	You must refrain from strenuous exercise for 48 hours prior to each assessment visit.

Caffeine	You are to refrain from drinking coffee (tea, cola drinks, chocolate) 24 hours before, and during, each study assessment visit.
Medication	Because of certain study restrictions, you should inform the study doctor or nurse if you are taking or are prescribed any medication during the study. They can decide in consultation with the prescribing doctor what action, if any, should be taken.

10. WHAT ARE THE POSSIBLE DISADVANTAGES AND RISKS OF TAKING PART?

If you have private medical insurance you should check with the company before agreeing to take part in the study to ensure that your participation will not affect your medical insurance.

If during the study a medical condition of which you were unaware is discovered, a full report will be sent to your GP (if you have no objections) and you will be advised of the appropriate course of action to take for treatment.

Study Procedures:

Questionnaires

The completion of questionnaires may take varying amounts of time, depending on the individual. The duration to complete the questionnaires has been listed in a table (question 6, page 3). You will be allowed breaks and the questionnaires will be staggered across the study visits to minimise distress to you.

Magnetic Resonance Imaging (MRI)

MRI is a standard hospital scanning technique that is able to take high definition pictures of the structure of your brain. There are no known side-effects of MRI scanning. It does not use radiation or drugs. We will have to ensure that you do not have metal or surgically inserted equipment in your body (e.g. cardiac pacemaker). For this reason we will ask you a detailed series of questions before bringing you into the scanner room. The MRI scanner is also quite confined and very noisy and you will need to lie very still when in the scanner, so scanning would not be a good idea if you have a problem with claustrophobia or with lying on your back for prolonged periods. It takes approximately 1 hour to complete the scan.

Magnetoencephalography (MEG)

You will be positioned within a large helmet-like device in a special room that can measure the very weak magnetic fields produced as a normal consequence of activity in your brain. You are not exposed to external electrical or magnetic fields. The experiment will take from 60-90 minutes, including a preparation time of about 30 minutes. You will be asked to remove any metallic objects (e.g, earrings, belts, and possibly other items of clothing). Alternative clothing will be provided if any of your clothing is inappropriate.

The top of your head will simply be positioned within the helmet-like device and a handful of sensors will also be attached to your head with tape (which should not cause discomfort nor leave any mark) to determine exactly where your head is in the scanner, while your brain activity is recorded. You will be told when the scan starts, and to keep very still during the scan (a cushion might be put around your head to help you keep still). It is important that muscle activity, such as head and eye movements and eye blinks are minimised. You may be asked to perform a task while you are being scanned, such as tapping your fingers, or looking at pictures. After your scan you will leave the MEG room.

CANTAB attention and memory testing
This is a series of computerized tests designed to look at different aspects of cognition, like attention, learning and memory. This session will last about 1 hour and, there will be breaks for rest and refreshment if required.

Electroencephalogram (EEG)
The EEG measures the electrical activity of the brain, which is useful in studies of human thinking as well as conditions like depression and schizophrenia. EEG scanning is standard procedure in hospitals and research settings and has been used for over 50 years.

During the EEG test visit, you will wear a Lycra cap containing 32 EEG electrodes that records electrical activity of the brain. Other electrodes will be placed around the eyes to record eye movements. Breathing rate will be measured using a band around your chest and electrodes will be placed on your hand and arms to measure skin conductance and to assess the condition of your heart (electrocardiogram or ECG).
For the tests of attention learning and memory, you will be seated in front of a computer monitor and wear head-phones. In response to various commands you will be asked to press a left or right button. These tests require no previous knowledge or use of computers. The test, including electrode or cap placement, will take less than 1 hour.

There are no risks associated with recording brain electrical activity using the EEG. The attachment and removal of the electrode cap and electrodes usually involves little discomfort, although it will be necessary to rub the surface of the skin of the scalp and apply gel to ensure a good contact between electrodes and scalp. The electrodes are small pads that are taped (painlessly) to your skin and which conduct readings to a monitor. They will not pierce your skin nor are they capable of providing electric shocks.

11. WHAT ARE THE POSSIBLE BENEFITS OF TAKING PART?

You are not expected to gain any benefit from taking part in this study. However, the knowledge gained from this study will improve our understanding of cognition in schizophrenia or schizoaffective disorder and assist future development of drugs used to treat cognitive impairment in patients with schizophrenia or schizoaffective disorder.

Expenses and payments:

You will receive payment in recognition of the time and inconvenience involved in taking part in this study. You will be paid **£450** for completing the study or, if you leave the study before completion, a proportion of that sum related to the completed assessments. In addition, you will also be reimbursed for travel expenses.

The payment (known as an honorarium) will be given to you after the follow-up has been completed successfully. GSK pays basic rate tax on the honorarium payment that you receive. It is up to you to inform the Inland Revenue and Department Work and Pensions (DWP) about the honorarium payment so that any changes can be made to your benefits, if necessary.

12. WHAT HAPPENS WHEN THE RESEARCH STUDY STOPS?

Taking part in this study is entirely voluntary and you can withdraw from the study at any time without giving a reason. This will not affect your possible future participation in clinical trials.

The investigator or GSK can terminate your involvement in the study for any of the following reasons:

- If you don't follow the investigator's instructions
- Something serious happens to you, which may require treatment.
- The investigator decides it is in the best interest of your health and welfare to discontinue.

13. WHAT IF THERE IS A PROBLEM?

Any complaint about the way you have been dealt with during the study or any possible harm you might suffer will be addressed. Please see Section 18 on this form for further details.

14. WILL MY TAKING PART IN THE STUDY BE KEPT CONFIDENTIAL?

Yes. All the information about your participation in this study will be kept confidential. The details are included in Section 19.

15. CONTACT DETAILS:

If you have any questions about the study, you can contact Dr. Gary Peters, the on-call physician or any other member of the study team at the Clinical Unit Cambridge, Addenbrooke's Centre for Clinical Investigation (Tel: 01223 296001).

You will receive a yellow card recording the study reference number, treatment and emergency telephone numbers should you wish to contact a doctor outside normal working hours. You should

keep this card with you for the duration of the study. The site security staff will have the telephone number of medical staff should you experience problems outside normal working hours. If you experience an adverse event which you or your family consider is serious or life-threatening, dial 999 for an ambulance and do not delay treatment by attempting to contact the physician in charge of the study.

This completes Section 1 of the Information Sheet.
If the information in Section 1 has interested you and you are considering participation, please continue to read the additional information in Section 2 before making any decision.

Section 2: General study information

16. WHAT IF RELEVANT NEW INFORMATION BECOMES AVAILABLE?

Sometimes during the course of a research project, new information becomes available about the assessments, treatment or drug that are being studied. If this happens, we will tell you about it and discuss whether you want to or should continue in the study. If you decide to continue in the study you will be asked to sign an updated consent form.

17. WHAT WILL HAPPEN IF I DON'T WANT TO CARRY ON WITH THE STUDY?

Taking part in this study is entirely voluntary and you can withdraw from the study at any time without giving a reason. If you withdraw from the study, we will destroy all your identifiable samples, but we will need to use the data collected up to your withdrawal.

18. WHAT IF THERE IS A PROBLEM?

Complaints:
If you have a concern about any aspect of this study, you should ask to speak with the researchers who will do their best to answer your questions. You may contact Dr Gary Peters, the on-call physician or any other member of the study team at the Clinical Unit Cambridge, Addenbrooke's Centre for Clinical Investigation (Tel: 01223 296001).

In addition, you may also contact the Independent Complaints Advocacy Service on 0845 4561084 or go to the following website http://www.seap.org.uk/icas/

Harm:
If your health deteriorates significantly because of your taking part in this study, you will be paid compensation. How much to pay is worked out by looking at court judgements made at the time you were injured, to see what people with similar injuries were awarded.

We will provide compensation for any injury caused by taking part in this study in accordance with the guidelines of the Association of the British Pharmaceutical Industry (ABPI).

We will pay compensation where the injury probably resulted from:

- a drug being tested or administered as part of the trial protocol
- any test or procedure you received as part of the trial

Any payment would be without legal commitment.

We would not be bound by these guidelines to pay compensation where

- the injury resulted from a drug or procedure outside the trial protocol
- the protocol was not followed by you (the volunteer)

19. WILL MY TAKING PART IN THIS STUDY BE KEPT CONFIDENTIAL?

Who has access to medical and personal information collected during the study?
The opinion of your general practitioner may be sought regarding your suitability, and he/she will be informed that you are participating in the study. You should not be taking part in other drug studies.
If you decide to participate in the study, the study doctor and staff will collect medical and personal information about you as part of doing the study. People who work for or with GSK or regulatory authorities responsible for approving medicines, will have access to this information at the site in order to check that the study is done properly. GSK staff who see this information at the site will keep it confidential.

The study site will also transfer to other parts of GSK some of the information it collects, in a coded form. The information transferred will not include your name, initials, address, or other direct identifiers. It will be assigned a code number that only the site can connect back to your name. Your permission to the study doctor and staff to use this information or share it with GSK and others as described below for the study doesn't automatically end at a particular time.

Once the study is over, you will be given access to medical information about you that you are entitled to see. At any time, you may ask your study doctor to let you see your personal information, e.g. name and address and to correct it if necessary.

What will the other parts of GlaxoSmithKline do with the information it gets?
GSK may use the information that the study doctor provides (i.e. the coded information):

- By storing and analyzing it electronically to find out what this study is telling us
- By sharing it with regulatory authorities that approve new medicines, or with groups that check that research is done properly
- By publishing the results of the study (this will not include any information that directly identifies you)
- By sharing it as part of research with other companies or universities for the purpose of further understanding or developing this methodology and with other GlaxoSmithKline offices in this country and in other countries (including those outside the European Union). If the information is sent to another country, GSK will apply the same level of protection to your information, to the extent permitted by local law
- By using it to plan new studies or other types of research or other medical purposes related to the development of the drug or class of drug.

20. WHAT WILL HAPPEN TO ANY SAMPLES I GIVE?

Urine samples will be collected to establish whether you are eligible for the study (drug screening & pregnancy testing for females). Thereafter, the samples will be destroyed. The data relating to the study however will be kept for up to 15 years.

Samples will be 'coded'; they will be identified only by the study number and the number of the volunteer (plus appropriate detail on session, time and date to accurately identify it). Only staff who are involved in the direct clinical care of the volunteers and the medical monitor will have access to the codes that links the volunteer number with identifiable information.

21. WILL ANY GENETIC TESTS BE DONE?

No genetic tests will be performed

22. WHAT WILL HAPPEN TO THE RESULTS OF THE RESEARCH STUDY?

In the future, the results of this study will be reported in an internal study report accessed only by the sponsor and government regulatory authorities. Data may also be published in scientific journals or presented at scientific meetings. In either case you will only be identified by your subject number.

23. WHO IS ORGANISING AND FUNDING THE RESEARCH?

The study is organised and funded by GlaxoSmithKline.

24. WHO HAS REVIEWED THE STUDY?

The Research Ethics Committee is an independent body, which reviews all proposed studies in healthy volunteers on a regular basis. Its objective is to safeguard the interests of the volunteers.
This study was reviewed and given a favourable ethical opinion by the Cambridgeshire 3 Research Ethics Committee.

25. CONFIDENTIAL INFORMATION

The information and the materials that are given to you in relation to the study are confidential information belonging to GlaxoSmithKline and should be kept private. You can discuss this information in confidence with your doctor or friends and family to decide about taking part in this study and talking about your healthcare.

<u>Thank you for your time and please feel free to ask questions if you have any further queries.</u>
